# Evaluating carbon removal: Integrating technical potential with environmental, social, governance criteria, and sequestration permanence

**DOI:** 10.1016/j.isci.2024.111418

**Published:** 2024-11-19

**Authors:** Jan Mertens, Christian Breyer, Ronnie Belmans, Corinne Gendron, Patrice Geoffron, Carolyn Fischer, Elodie Du Fornel, Richard Lester, Kimberly A. Nicholas, Paulo Emilio V. de Miranda, Sarah Palhol, Peter Verwee, Olivier Sala, Michael Webber, Koenraad Debackere

**Affiliations:** 1 ENGIE Research, 1 PL. Samuel de Champlain, Paris-la Défense, 92930 Paris, France; 2 Department of Electromechanical, System and Metal Engineering, Ghent University, Technologiepark Zwijnaarde 131, Zwijnaarde, Belgium; 3 LUT University, Yliopistonkatu 34, 53850 Lappeenranta, Finland; 4 Electrical Energy and Computer Architectures, K.U.Leuven, Kasteelpark Arenberg, 3001 Leuven, Belgium; 5 EnergyVille, Thor Park 8310, 3600 Genk, Belgium; 6 Université Du Québec à Montréal (UQAM), Département de Stratégie, Responsabilité Sociale et Environnementale, École des Sciences de La Gestion (ESG), Québec, Canada; 7 Dauphine Economics Laboratory, Université Paris Dauphine-PSL, Place Du Maréchal de Lattre de Tassigny, 75016 Paris, France; 8 Sustainability and Infrastructure Team, World Bank Group, 1818 H Street, NW Washington, DC 20433, USA; 9 Department of Nuclear Science and Engineering, Massachusetts Institute of Technology (MIT), Cambridge, MA, USA; 10 Lund University Centre for Sustainability Studies (LUCSUS), Lund University, 221 00 Lund, Sweden; 11 Hydrogen Laboratory at Coppe-Federal University of Rio de Janeiro, Av. Moniz Aragão, 207, Rio de Janeiro 21941-594, Brazil; 12 Engie Impact, Simon Bolivarlaan 34 1000 Brussel, Belgium; 13 Department of Mechanical Engineering, The University of Texas at Austin, 204 E. Dean Keeton St, Stop C2200, Austin, TX 78712-1591, United States; 14 KU Leuven, ECOOM, Department of Managerial Economics, Strategy and Innovation, Faculty of Economics and Business, Naamsestraat 69, BE-3000 Leuven, Belgium

**Keywords:** Energy resources, Energy policy, Energy sustainability, Energy systems

## Abstract

Climate modeling suggests that achieving international climate goals requires a reduction in current CO_2_ emissions by over 90%, with any remaining emissions to be addressed through carbon dioxide removal (CDR) solutions. Sixteen CDR strategies are evaluated by integrating technical potential, environmental, social, and governance (ESG) criteria, along with sequestration permanence. This evaluation, conducted by ENGIE’s scientific council using an interdisciplinary Delphi panel methodology, proposes a “quality” measure for each technology. This measure combines ESG scores and sequestration timescales to rank and select the most promising solutions. The findings highlight the necessity for further research to understand and mitigate ESG impacts, aiming to inform both future research and current decision-making to support the effective and legitimate use of CDR strategies.

## Introduction

**Figure 1:**
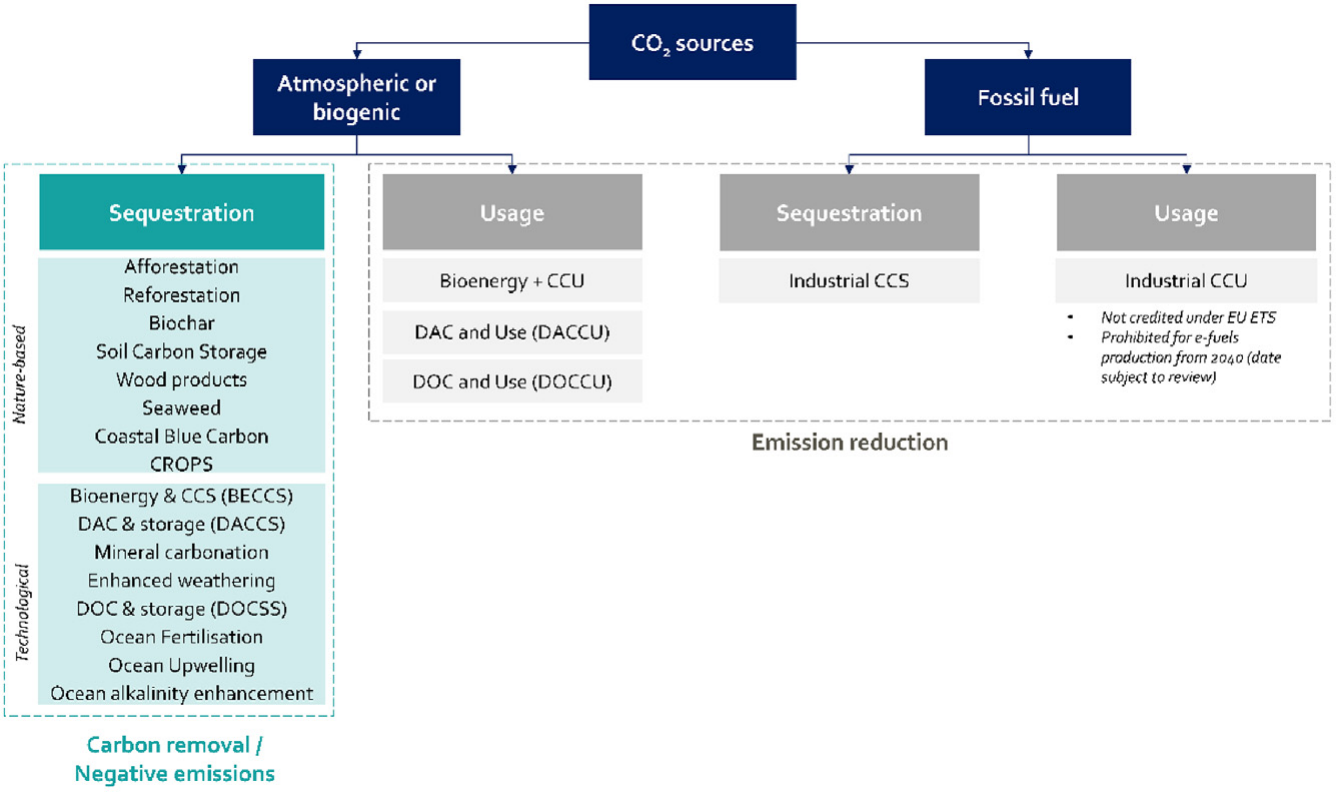
Potential pathways for carbon capture and sequestration (CCS), carbon capture and utilization (CCU), and carbon removal Two dimensions structure the technological landscape of CO_2_ management technologies: biogenic versus fossil CO_2_ and CO_2_ sequestration versus use. CCS of fossil fuel-based CO_2_ and CCU both allow emission reduction while CDR can only be achieved through the sequestration of biogenic or atmospheric CO_2_.

Climate modeling studies demonstrate that to reach internationally agreed climate ambitions, the first and foremost focus should be on the reduction of current CO_2_ emissions estimated at 40.7 Gtonnes in 2023^1^ by more than 90%.^2^^,^^3^ In February 2024, the European Commission presented its assessment for a 2040 climate target for the European Union (EU) that suggested reducing the EU’s net greenhouse gas emissions with 90% by 2040 relative to 1990. Within the 90% emission reductions, both carbon capture and utilization (CCU) and carbon capture and sequestration (CCS) will play a role, where CCU can use carbon as a resource to supply essential processes where high energy density, hydrocarbon feedstocks, or long-term energy storage are crucial,^4^^,^^5^^,^^6^ and CCS may be used in industrial sectors that are hard to electrify or for which molecules only solve part of the challenge, such as cement production (Figure 1). Carbon dioxide removal (CDR) solutions,^7^^,^^8^^,^^9^ which can be either nature-based or technological approaches to take CO_2_ out of the air and sequester it, will be required to compensate for the remaining Gtonnes of yearly CO_2_ emissions.^2^^,^^3^ This classification into nature-based and technological solutions is not absolute^10^ since quite some technological solutions rely on nature to store CO_2_ (e.g., enhanced weathering, ocean alkalinization, bioenergy CCS, etc.) and similarly for some nature based solutions, enabling technologies need to be deployed at large scale (e.g., biochar, soil carbon sequestration, etc.) There is little agreement on the relative costs and benefits of potential CDR measures, particularly from a holistic sense including their social, environmental, and economic impacts. Technically, only the emissions of cement production may remain from the limestone input, while all other fossil emissions can be entirely replaced by non-fossil solutions.^11^

The potential and major challenges related to the deployment at scale of the 16 carbon removal strategies are presented in Figure 1 and Annex 1. These are the most common CDR options that are today either being studied or already deployed, but the list is not exhaustive. Annex 1 provides a brief description for each of them including the following.

(1) Current estimated technology readiness level (TRL).
(2) Technical potential in Gtonnes of CO_2_ removed per year.
(3) Estimated cost range in 2030.
(4) The cost trend toward 2050.
(5) The main environmental, social, and governance (ESG) dimensions involved.

Here we use these elements to develop a method to assess the quality of CDR strategies and provide a ranking and evaluation of sixteen strategies to inform both research and practice. This interdisciplinary assessment was conducted by ENGIE’s scientific council, authoring this paper and consists of members who combine academic and industrial expertise in research and development management, energy, environmental economics and policy, sociology, and sustainability and climate change. This implies that this work represents an expert appraisal from people with broad relevant scientific backgrounds. This ensures an interdisciplinary approach while the fact that the experts come from across the globe ensures its international character.

The scientific council adopted a Delphi panel methodology to converge on a consensus assessment of the potential, the feasibility and the impact of the array of CDR shown in Figure 1. The Delphi processes ran throughout a full year, starting with the questions addressed by ENGIE’s Executive Committee to the scientific council: “What are the critical technologies related to CO_2_ removal in the energy transition? What is their potential and the major challenges relating to their deployment at scale? What is the potential merit order of the technological systems that could emerge?” To address this, an initial two-day face-to-face meeting was convened in Paris in May 2023, bringing together startups, academics, oil and gas companies, and research ecosystems for presentations, analyses and data-driven insights on the topic of CDR, followed by Q&A sessions with council members. Between May 2023 and January 2024, several online discussions were held with council members, both in group settings and one-on-one, to further develop the inquiries. Individual insights were integrated along the Delphi pathway toward building consensus, resulting in a matrix of insights based on the criteria vector. A final two-day face-to-face meeting was held in January 2024 in Boston, where the study’s outcomes were discussed and ultimately approved and supported by all council members based on the most recent insights available from scientific studies. This culminated in a deliverable presented to ENGIE’s Executive Committee in March 2024.

### The technical volumetric potential of CDR options largely exceeds the required removal

**Figure 2:**
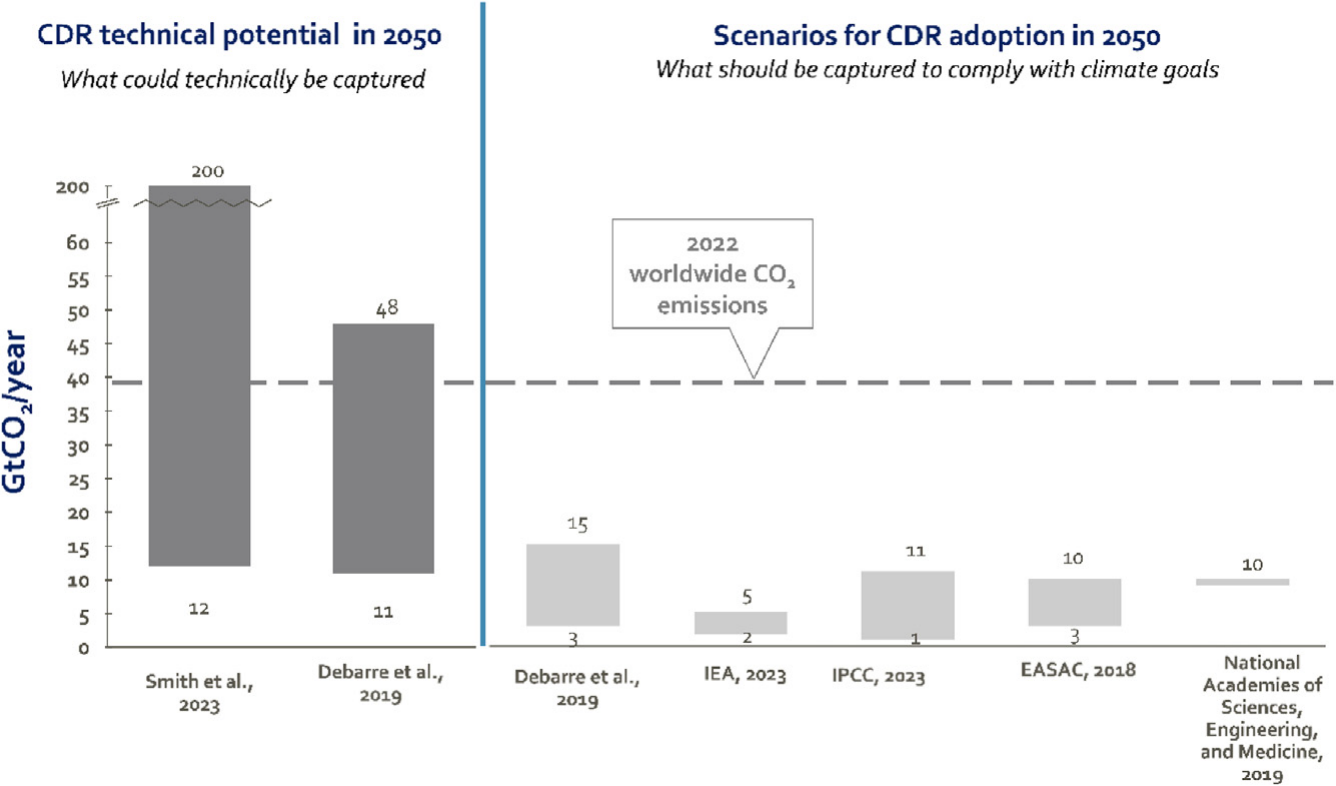
Estimated technical potential of carbon dioxide removal (CDR) options (left part of the figure) versus their likely uptake in different climate modeling scenarios (right part of the figure) in Gtonnes per year by 2050 The dotted line shows today’s yearly CO_2_ emissions.

Reducing our current emissions (around 40 Gtonnes) with 90%^2^^,^^3^ would leave around 4 Gtonnes of CO_2_ to be removed by CDR. The Sixth Assessment Report (IPCC, 2023) estimates that the CDR demand beyond mid-century could be as high as 15 Gtonnes of CO_2_ per year. Going beyond and up to the safe and just planetary climate boundary of 1.0⁰C^12^, this would lead to a CDR demand of up to 40 Gtonnes of CO_2_ per year.^13^ Figure 2 (left) presents the estimated range of technical CDR potential expressed in Gtonnes of CO_2_ of two studies: (1) *Smith* et al.^9^ who estimate the technical potential between 12 and 200 Gtonnes per year and (2) *Debarre* et al.^14^ who are more conservative and present a range between 11 and 48 Gtonnes per year. Both exceed by far the required 4 Gtonnes per year and thus technically speaking, it is possible to remove the remaining CO_2_ emissions in the 1.5°C scenario by 2050. The question remains how much of this “potential” will be deployed. This will depend on many parameters such as cost, ESG risks, timescale of CO_2_ sequestration, and (local) social acceptance^15^ of these technologies. Figure 2 (right) presents five different roadmaps toward carbon neutrality and the adoption of CDR expected in these roadmaps. A lot of uncertainty exists and the results depend highly on the modeling hypotheses. *IPCC*^2^ shows a range as low as 1 Gtonne and up to 11 Gtonnes per year while *Debarre* et al.^14^ show a value as high as 15 Gtonnes per year. Recent Copernicus data show that the average global surface temperature already temporarily exceeded the 1.5°C threshold for 12 consecutive months from July 2023 to June 2024, further adding to the necessity of CDR deployment.

The dashed line in Figure 2 shows the current annual CO_2_ emission level and illustrates how the technical potential of CDR options considered exceeds today’s emissions. However, even though deploying CDR at this scale could avoid fossil fuel stranded assets, we must ensure CDR is not being developed and deployed at large scale to continue fossil fuel use. The combination of emissions reduction (<90%) first and emission removal for the last 10% of emissions is the scientifically sound way to reach the ambitious climate targets, whereas additional CDR demand arises due to additional carbon removal to meet the 1.5°C target.^2^^,^^3^

### A wide variety in “quality” of CDR technologies exists based on their ESG score and permanence

We assessed the quality of CDR based on two factors: their risks to ESG sustainability and the timescale of CO_2_ sequestration (permanence). First, ESG risks exist for each technology, not only for CDR options. However, for CDR many unknowns still exist for various technologies and thus their ESG impact is not yet well understood.^16^^,^^17^^,^^18^^,^^19^ ESG impact will however be a crucial parameter determining their acceptance. The ESG aspects involved in each technology were extensively discussed among the authors as well as with experts working on these technologies. This discussion led to an ESG score between 1 (worst) and 5 (best) given to each of the 16 technologies (Table 1). Two CDR options received a maximum score of 5: reforestation, i.e., restoring forests in areas where there was previously forest in the last 50 years, and using wood for construction. In contrast, many of the ocean-based technologies, such as ocean fertilization, ocean upwelling, ocean alkalinity enhancement and CROPS, were given the lowest value of 1, mainly due to the many uncertainties and unknown impacts of these technologies on ocean marine life and biodiversity, as seen in current scientific debate on amongst others deep sea mining. However, the technical potential of many of these ocean technologies for sequestering CO_2_ is significant. Therefore, an important area of future research is to better understand the negative ESG impacts of ocean-based CDR and then evaluate whether and how they can be reduced.^20^ In addition to the ESG factors for each technology, the “quality” of a CDR option is associated not just with its ESG attributes but also with the permanence for which the technology sequesters CO_2_.^21^ Again, a well-informed and intensely debated expert opinion on this timescale was estimated for each technology and is presented in Table 1. A quality score is then calculated, integrating the ESG score as well as the sequestration timescale (expected sequestration time of CO_2_) of the solutions. As a result of the discussion during the Delphi process, the ESG score is given a slightly higher weight than the sequestration timescale. The reasoning being that the climate crisis must be solved urgently, i.e., within the next 3 decades. Therefore, assuming all CDR have at least this 30 years sequestration timescale (e.g., wood for construction, afforestation, reforestation, etc.), the ESG impact of CDR was esteemed more important and given slightly more weight than the sequestration timescale. The final “quality” score for each of the 16 CDR options is presented in Table 1 and reveals that direct air carbon capture and sequestration (DACCS) is attributed the highest “quality” closely followed by reforestation, wood for construction, and biochar.

**Table 1:** The expert appraisal of each of the CDR options’ ESG score as well as their expected CO_2_ sequestration timescale

	ESG Score (1–5)	Sequestration timescale (years)	“Quality”(ESG+sequestration timescale)
Direct air carbon capture and sequestration(DACCS)	4	>1,000	9
Wood for construction	5	<100	8.5
Reforestation	5	<100	8.5
Biochar	4	100–1,000	8
Mineral carbonation	3	>1,000	7.5
Enhanced weathering	3	>1,000	7.5
Direct ocean carbon capture and storage (DOCCS)	3	>1,000	7.5
Afforestation	4	<100	7
Soil carbon storage	4	<100	7
Seaweed	3	100–1,000	6.5
Bio energy carbon capture and sequestration (BECCS)	2	>1,000	6
Costal blue carbon	3	<100	5.5
Ocean fertilization	1	100–1,000	3.5
Ocean upwelling	1	100–1,000	3.5
Ocean alkalinity enhancement	1	100–1,000	3.5
Crops	1	100–1,000	3.5

Based on these two parameters, a “quality” measure is calculated given more weight to ESG as compared to CO_2_ sequestration timescale: “Quality” = ESG score ∗ 1.5 + CO_2_ timescale value (whereby a timescale value of 1 was given for a CDR options with an expected CO_2_ sequestration time <100 years, a value of 2 for a timescale between 100 and 1,000 years and a value of 3 for a timescale >1,000 years).

### Integrating technical potential, ESG criteria, permanence, and TRL of CDR options to select most promising solutions

**Figure 3:**
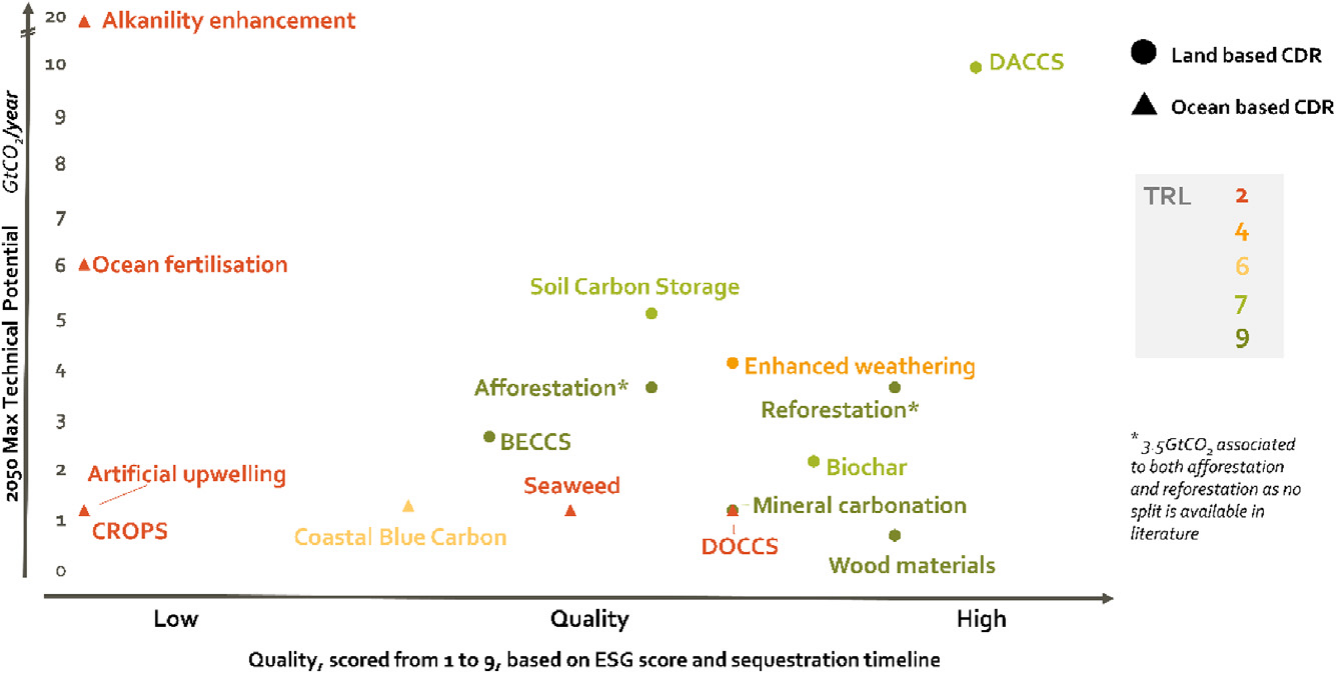
“Quality” of CDR options versus their 2050 technical potential in Gtonnes of CO_2_ per year Estimated “quality” is based on their ESG score and the estimated CO_2_ sequestration timescales. The color indicates the TRL of each technology, ranging from low (red) to high (green).

Figure 3 presents the estimated technical potential of each of the CDR solutions in Gtonnes of CO_2_ per year in the year 2050 covered in the study versus its “quality.” On top, today’s TRL of each technology is plotted as a color spectrum from red (TRL = 2) to green (TRL = 9). This allows to select the most promising technologies with respect to individual preferences. A company ready to invest today will only consider solutions with a high TRL value and meeting the quality it is looking for vis-a-vis its stakeholders. Other companies building roadmaps for their carbon neutrality targets and may select a few different technologies with a varying TRL level as a function of when the CDR technology will need to be rolled out.

Although cost was not taken into account in this study, it will play an important parameter in the selection of CDR that will be adopted. Therefore, Annex 1 presents the estimated cost variances of the different CDR options in 2030 and how they are expected to evolve toward 2050. The estimated cost ranges are very large and, in some cases, too large to reach to a well-informed decision today.

It is important to remind again the required amount of CO_2_ removal by 2050 is estimated close to 4 Gtonnes. This implies that even technologies such as biochar (2 Gtonnes) or wood materials (0.4 Gtonnes), which seem to be low positioned in Figure 3, can be significant and thus important to have in the CDR portfolio to be developed by 2050.

### Conclusion

The potential and challenges associated with large-scale deployment of CDR options have been evaluated by an interdisciplinary group of experts. Utilizing the Delphi approach, which seeks to achieve consensus among participating experts, the estimated technical potential of these technologies largely exceeds the required emission removal efforts by 2050, with the estimated range of the technical CDR potential between 11 and 200 Gtonnes per year, far exceeding the estimated 4 Gtonnes required to be removed using CDR to reach ambitious climate targets, given the assumptions considered.

A wide variety in the “quality” of CDR technologies exists based on their ESG score and CO_2_ sequestration timescale. Today, there is a discussion on whether the voluntary carbon market is supporting effective climate mitigation or not as recently demonstrated by *Trencher* et al.^22^ This study therefore proposes a “quality” measure for each technology based on a consensus expert opinion, integrating the ESG score and the sequestration timescale, allowing the ranking and selection of the most promising solutions tailored to individual/company preference. We see a need for increased scientific research on all technologies to better understand and quantify their ESG impacts. Consequently, it should be evaluated whether and how these ESG impacts can be reduced. Ensuring that CDR options are utilized as a supplementary measure to drastic emissions reductions, rather than to prolong the use of fossil fuels, is crucial. CDR technologies should be considered once efforts have already achieved a >90% reduction in emissions. Here we have developed a method to assess the quality of carbon removal technologies and applied it to rank 16 technologies available today, which we hope will inform both future research and current decision making to support limited, effective, legitimate, and appropriate use of carbon removal strategies.

## Declaration of interests

The authors declare no competing interests.
