# Supplementary material for: Evaluating carbon removal: Integrating technical potential with environmental, social, governance criteria, and sequestration permanence
Source: iScience. 2024 Nov 19;27(12):111418. doi: 10.1016/j.isci.2024.111418 (PMC11664158; doi:10.1016/j.isci.2024.111418)
Supplement: Document S1. Figures S1–S4 [file mmc1.pdf]

iScience, Volume *27*

Supplemental information

Evaluating carbon removal: Integrating technical potential with environmental, social, governance criteria, and sequestration permanence

Jan Mertens, Christian Breyer, Ronnie Belmans, Corinne Gendron, Patrice Geoffron, Carolyn Fischer, Élodie Du Fornel, Richard Lester, Kimberly A. Nicholas, Paulo Emilio V. de Miranda, Sarah Palhol, Peter Verwee, Olivier Sala, Michael Webber, and Koenraad Debackere

Fig S.1 Carbon Dioxide Removal (CDR) Technologies in this evaluation

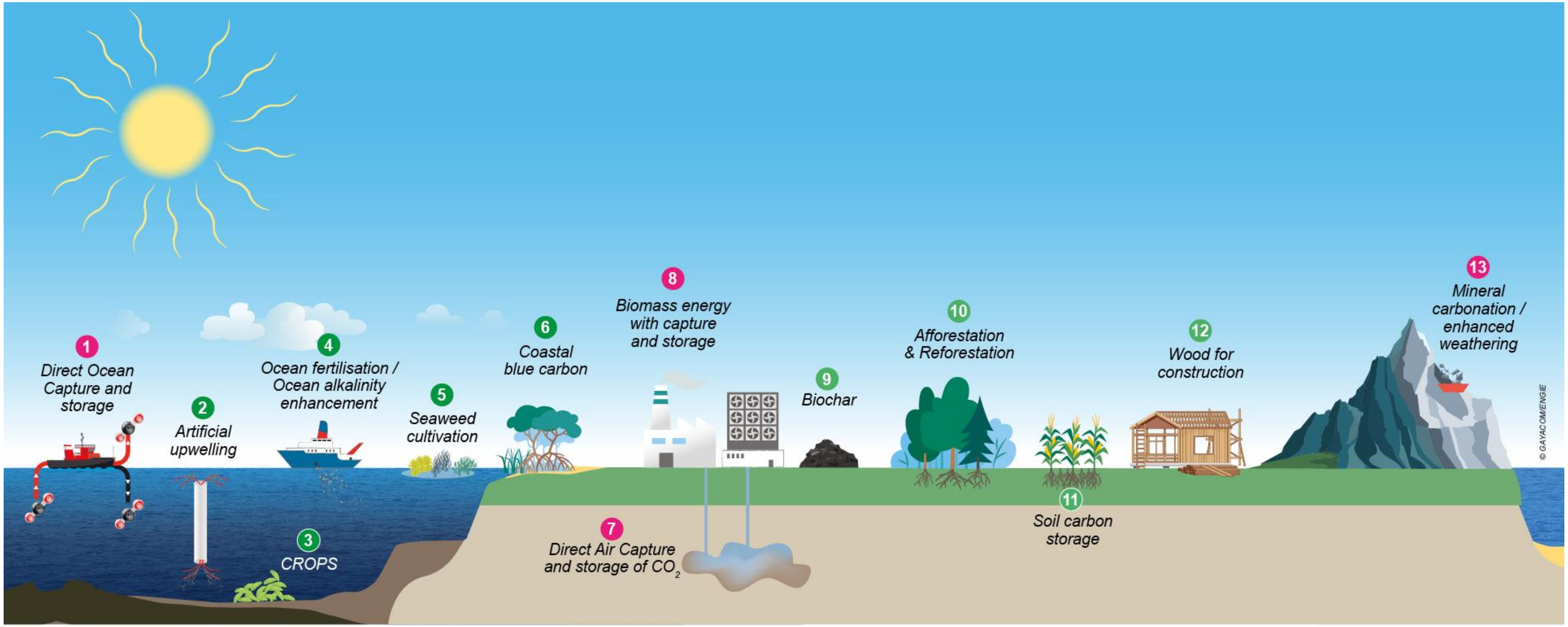

Carbon Dioxide Removals (CDR technologies)

1. **Direct Ocean Capture and storage**
2. **Artificial upwelling**
3. **CROPS**
4. **Ocean fertilisation / Ocean alkalinity enhancement** *(Incressing population of carbon-absorbing plankton)*
5. **Seaweed cultivation**
6. **Coastal blue carbon** *(Reforestation/ Mangrove restoration)*
7. **Direct Air Capture and storage of CO_2**
8. **Biomass energy with capture and storage** *(Using biomass for energy and capturing the CO_2)*
9. **Biochar** *(Carbon-rich charcoal from burnt crops added to soil)*
10. **Afforestation & Reforestation** *(Planting vast forests)*
11. **Soil carbon storage**
12. **Wood for construction**
13. **Mineral carbonation / enhanced weathering**

Legend:
- *Nature based*
- *Technology based*

Fig S.2 Technology-based Carbon Dioxide Removal technologies in this evaluation

	Direct Air Carbon Capture and Sequestration (DACCS)	Bio Energy Carbon Capture and Sequestration (BECCS)	Mineral Carbonation	Enhanced Weathering
**Definition**	CO_2 is captured from the atmosphere and then sequestered underground	CO_2, produced from biomass transformation into energy, is captured and then sequestered underground	conversion of alkaline minerals to solid carbonates, used as construction or filling materials	alkaline minerals spread on the ground to increase the speed of naturally occurring weathering of silicates minerals
**Maturity** *(TRL)*	7	7-9	7-9	4
**Potential 2050** *(GtCO$_2$/y)*	10	2.5	1	4
**Cost 2030** *(€/tCO$_2$)*	100-600	100-200	50-600	50-200
**Cost trend 2050**	↘	↘	↘	↘
**Environmental, social, governance aspects**	Low biodiversity impact<br>Social acceptance risk<br>Lack of regulations	Negative effect on biodiversity<br>Social acceptance risk	Mining needed (energy intensive)<br>Possible restrictions on uses,<br>Excluded from Emission Trading Systems	Increase soil quality, fight ocean acidification,<br>Mining needed (energy intensive)<br>No regulations

Fig S.3 Onshore nature-based Carbon Dioxide Removal technologies in this evaluation

	Reforestation	Afforestation	Soil Carbon Storage	Biochar	Wood for construction
**Definition**	Planting forest on land that used to be forest (<50 years)	Planting forest on lands that used to be grasslands or shrublands	Increase of soil organic carbon content through agricultural management practices	Stabilize biomass into a recalcitrant char than can be used as soil amendment	Use wood for buildings
**Maturity** *(TRL)*	9	9	7	7	9
**Potential 2050** *($GtCO_2/y$)*	3.5*	3.5*	5	2	0.4
**Cost 2030** *($€/tCO_2$)*	5-50	5-50	0-100	30-120	Low
**Cost trend 2050**	↗	↗	↗	↘	↘
**Environmental, social, governance aspects**	Conservation of biodiversity, Prevent deforestation	Fight desertification<br>Possible negative effect on biodiversity	Increase soil quality<br>Monitoring, Reporting, Verification (MRV) complex<br>Possible limitation on amount of offset	Increase soil quality<br>Risk for food security<br>Possible restrictions on applications	Avoid cement emissions<br>Europe favours usage of wood for construction

**Total of 7$GtCO_2$ potential for afforestation & reforestation has been split equally between both solutions as no better info is available*

Fig S.4 Ocean-based Carbon Dioxide Removal technologies in this evaluation

	Coastal Blue Carbon	Direct Ocean Carbon Capture and Sequestration (DOCCS)	Ocean Fertilization	Ocean artificial upwelling	Ocean alkalinity enhancement	Seaweed cultivation	Crops
**Definition**	Carbon captured and stored by coastal ecosystems	Captures CO_2 directly from the seawater	Addition of micronutrients to increase CO_2 uptake	Bring nutrient-rich seawater from deep ocean to the surface	Enhanced weathering applied to the ocean	Large-scale seaweed cultivation and sequestration	burial of crop residues in the deep ocean
**Maturity** *(TRL)*	6	Low	Low	Low	Low	Low	Low
**Potential 2050** *($GtCO_2/y$)*	1	1	6	1	20	1	1
**Cost 2030** *(€/tCO_2)*	170-220	100-350	50-500	High	40-260	25-125	50-100
**Cost trend 2050**	↘	↘	↘	↘	↘	↘	↘
**Environmental, social, governance aspects**	Conservation of biodiversity<br>Monitoring, Reporting, Verification (MRV) risk<br>Green washing complaints<br>Dependency on national regulation	Fight ocean acidification<br>Potential impact on marine ecosystem	Change in natural cycles,<br>MRV risks,<br>May violate London Convention of Seas	Disturbance to regional balances,<br>MRV risks<br>Ocean's responsibility legal issues	Fight ocean acidification<br>MRV risk,<br>Disturbance of ocean biogeochemical functioning<br>May violate London Convention of Seas	Unknown potential on marine ecosystem,<br>MRV risk<br>Disposable of valuable, nutritional and biomaterial resources<br>May violate London Convention of Seas	Potential impact on marine ecosystem,<br>MRV risk<br>Disposable of valuable, nutritional and biomaterial resources<br>May violate London Convention of Seas
